# Facile Self-Assembly of Exfoliated Graphene/PANI Film for High-Energy Zn-Ion Micro-Supercapacitors

**DOI:** 10.3390/molecules28114470

**Published:** 2023-05-31

**Authors:** Yili Wang, Jin Niu

**Affiliations:** Laboratory of Electrochemical Process and Technology for Materials, State Key Laboratory of Chemical Resource Engineering, Beijing University of Chemical Technology, Beijing 100029, China

**Keywords:** micro-supercapacitor, Zn anode, EG/PANI film, facile preparation

## Abstract

The Zn-ion micro-supercapacitor (ZMSC) is a promising candidate for developing miniaturized and integrated energy storage devices. To achieve high-performance functional groups with simple processing to composite with rod-like active PANI fibers, we prepared exfoliated graphene (EG) with an appropriate amount of O-containing functional groups. The appropriate O content simultaneously facilitated self-assembly of the EG and PANI fibers and maintained the electric conductivity of the composite, producing a free-standing EG/PANI film without additional conductive additives or current collectors. As an interdigital electrode for the ZMSC, the EG/PANI film showed ultrahigh capacitance of 1.8 F cm^−2^ at 2.6 mA cm^−2^ (361.3 F g^−1^ at 0.5 A g^−1^) and landmark energy density of 755.8 μWh cm^−2^ at 2.3 mW cm^−2^ (148.2 Wh kg^−1^ at 451.7 W kg^−1^). The facile preparation of the high-performance EG/PANI electrode provides a potential path for practical applications with ZMSCs.

## 1. Introduction

For the development of miniaturized and integrated energy storage devices, micro-supercapacitors (MSCs) have aroused much attention due to their small size, rapid electron-ion kinetics, designable patterns, etc. [1]. In principle, MSCs are composed of positive and negative tandem electrodes. The capacitance of the MSC ($C_{\mathrm{MSC}}$) is dependent on the performance of both positive and negative electrodes, which can be expressed as [2]:

(1) $$C_{\mathrm{MSC}}=\frac{f_{n}\times C_{n}\times V_{n}}{\left(f_{p}+f_{n}\right)\times \left(V_{p}+V_{n}\right)}=\frac{f_{p}\times C_{p}\times V_{p}}{\left(f_{p}+f_{n}\right)\times \left(V_{p}+V_{n}\right)}$$

where $f_{n}$, $f_{p}$, $C_{n}$, $C_{p}$, $V_{n}$, and $V_{p}$ denote the fraction, specific capacitance, and potential window of the negative and positive electrodes, respectively. Since the negative electrode usually has a lower capacity than the positive electrode, the theoretical energy density of the device is generally limited by $\frac{1}{2}C_{n}\times V_{n}\times \left(V_{p}+V_{n}\right)$ no matter how high the $C_{p}$ achieved [2]. Taking the widely used negative electrode material active carbon as an example, it usually shows a *C_n_* of ca. 200 F g^−1^ [3]; thus, the energy density of the assembled device is limited to 50 Wh kg^−1^ in aqueous electrolyte, even if *C_p_* reaches the high value of ca. 3000 F g^−1^ [4]. Therefore, as energy storage devices, developing high-capacitance negative electrodes is significant for high-performance MSCs.

Recently, Zn metal has been proposed as a good candidate for high-performance negative electrodes due to the following advantages. Firstly, it has a large capacity of 823 mAh g^−1^ and a low redox potential of −0.76 V (Zn^2+^/Zn, vs. SHE), which can provide high capacitance and a high potential window [5]. In addition, Zn metal is stable, naturally abundant, and non-polluting. Moreover, Zn metal can work safely in aqueous solution compared with other metal cations (such as Li^+^, Na^+^, K^+^, Mg^2+^, Ca^2+^, and Al^3+^) [6]. Therefore, Zn-ion MSCs (ZMSCs) have the potential for use in high-performance hybrid MSCs with high capacitance and energy densities for practical applications [7]. Since they moved into the spotlight in 2018, ZMSCs have received increasing attention as promising energy storage devices [8]. To match the Zn negative electrodes, a variety of positive electrode materials (such as porous carbon material [9], metal oxide [10], MXene [11], conducting polymer [12], etc.) have been used for the positive electrodes. For example, Tang et al. [6] used activated carbon sheets as the positive electrode to match the Zn foil, which exhibited a capacity of 120 F g^−1^ and energy density of 52.7 Wh kg^−1^ (at a power density of 1725 W kg^−1^). Zeng et al. [13] prepared the RGO-MXene composite anode, which achieved high energy densities of 128.6 F g^−1^ and 37.9 Wh kg^−1^ (at 279.9 W kg^−1^).

Despite the significant achievements that have been made, some challenges still remain, mainly the limited device performance and the complex preparation method for ZMSCs. From the perspective of the device performance, it is still essential to increase the energy and power densities based on the total electrodes rather than the active materials. However, the device performance from most previous work was limited due to the inactive components of the electrodes. Specifically, the electrodes usually contain some ineffective components, including conductive additives and binders (~20 wt% of the active materials), current collectors (with a weight ratio several times higher than the active materials), etc. [14]. Various approaches have been proposed for the preparation method for ZMSCs, such as photolithography, laser writing, evaporation, electrodeposition, etc. [15]. However, these processes usually have disadvantages, such as complex procedures, time consumption, high cost, etc. [16]. Therefore, it is still necessary to develop a method for preparing ZMSCs that allows high device performance and simple processing.

In this context, we employed modified exfoliated graphene (EG) with an appropriate amount of O-containing functional groups and rod-like PANI fibers to prepare an EG/PANI composite electrode to achieve the above two goals. The appropriate O content facilitates the self-assembly of EG and PANI, producing free-standing film electrodes without additional binders within the electrodes [17]. In addition, the limited O content can maintain good electric conductivity without any further treatment, providing a large-scale and fast method [18]. Moreover, the rod-like PANI fibers can be effectively dispersed and inserted in the 2D graphene layer, which can induce various active sites in the electrodes while maintaining their mechanical strength. Furthermore, the film can be easily processed into a designed pattern, thus simultaneously enabling high device performance and simple processing with ZMSCs.

## 2. Results and Discussion

**Figure 1:**
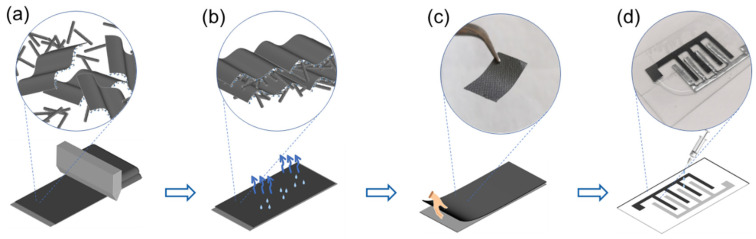
Scheme for the preparation of ZMSCs. (**a**) Casting the solution of EG and PANI. (**b**) Room-temperature evaporation of the EG/PANI composites. (**c**) Detaching the free-standing EG/PANI film from the substrate. (**d**) Construction of ZMSCs with designed EG/PANI and Zn patterns.

To fabricate the EG/PANI electrodes, we first mixed EG and PANI using facile magnetic stirring. EG was prepared using a modified chemical exfoliation method. The appropriate O content and thickness for the EG facilitated the dilution of graphene sheets and the self-assembly of the EG and rod-like PANI (Figure 1a). Due to the good electric conductivity of EG, a compact and dense film was directly obtained after room-temperature evaporation without an additional chemical reduction process (Figure 1b). Then, the self-assembled film could be detached from the substrate to form a free-standing electrode (Figure 1c). After that, the free-standing film electrode could be simply cut into designed patterns (such as interdigital patterns, as in our work) via laser or mechanical scribing that could be further used to build ZMSCs with designed Zn patterns (Figure 1d). Traditional preparation methods usually need additional reduction and purification processes under specific conditions [19]. Here, the process for the EG/PANI-based ZMSCs showed obvious superiorities due to the unique composition, free-standing ability, and facile electrode patterning of the EG/PANI film.

**Figure 2:**
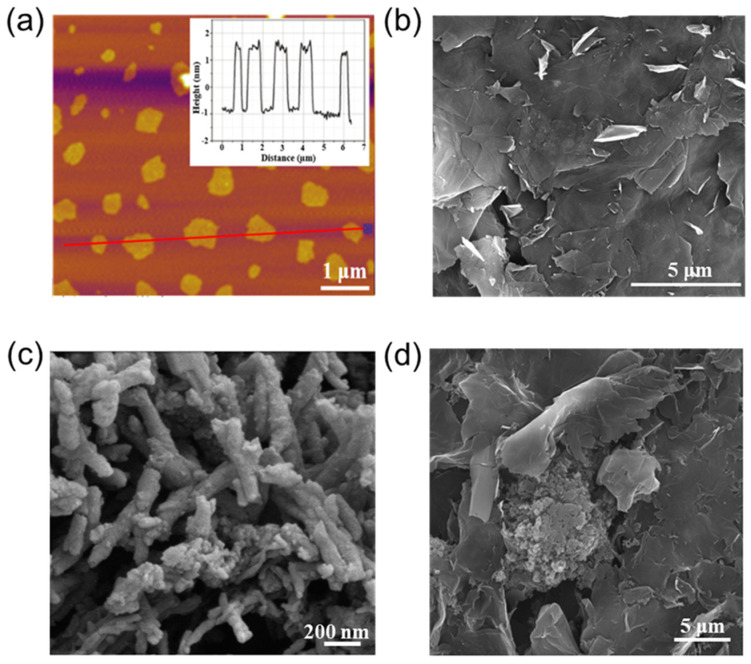
(**a**) AFM images of EG; inset is the height profile. (**b**–**d**) SEM images of EG, PANI, and EG/PANI.

We subsequently performed AFM and SEM measurements to investigate the morphology of the EG/PANI composite. As shown in Figure 2a, the height of the EG was ca. 2 nm, confirming that most of the EG nanosheets were composed of 3~5 graphene layers [20]. The SEM image showed that the size of the EG nanosheets was several micrometers and they had few wrinkles and incompact pores (Figure 2b), indicating the high quality of the EG sheets. In addition, it was found that the PANI appeared as rod-like tubes hundreds of nanometers in length (Figure 2c). After employing the self-assembly casting method, the PANI was well-inserted into the matrix of the EG sheets (Figure 2d). Since EG has good electroconductivity, it can act as a conductive matrix. In addition, PANI with rich N-containing functional groups can provide efficient active sites for ionic storage, enhancing the capacitive performance of the electrodes for capacitors. Thus, the EG/PANI film would be beneficial for efficient electron transfer, charge diffusion, and storage at the same time [21].

**Figure 3:**
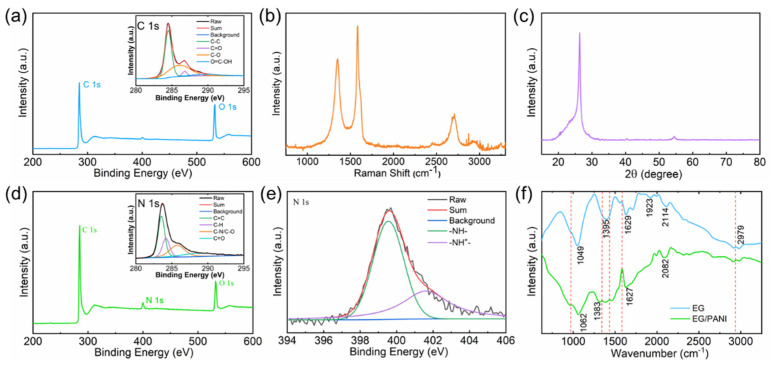
(**a**) XPS spectrum of EG (inset: high−resolution C 1s XPS spectrum of EG). (**b**) Raman spectrum of EG. (**c**) XRD pattern of EG. (**d**) XPS spectrum of EG/PANI (inset: high−resolution C 1s XPS spectrum of EG/PANI). (**e**) High−resolution N 1s XPS spectrum of EG/PANI. (**f**) FTIR patterns of EG and EG/PANI.

In order to further evaluate the microstructures of the electrodes, we performed XPS measurements to calculate the atomic components of the EG/PANI film. It was found that the XPS spectrum of EG mainly exhibited C and O peaks before composition. The O content was calculated to be ca. 14.9% (Figure 3a). As shown in the inset of Figure 3a, the deconvolutions of the C 1s spectrum were mainly located at 286.8 eV (corresponding to the epoxy and hydroxyl groups), 288.5 eV (attributed to the carbonyl peak), and 291.3 eV (resulting from the carboxyl group) [22]. The XPS result indicated that EG had a lower O content than graphene oxide but a similar O bonding structure, allowing it to facilitate dispersion and maintain the electric conductivity of the EG at the same time [23]. Raman and XRD measurements were also performed to evaluate the microstructure of EG. As shown in Figure 3b, there were two obvious peaks around ~1580 cm^−1^ and ~1350 cm^−1^. The former was the D peak, which is attributable to the E2 g vibration mode present in sp^2^-bonded graphitic carbons. The latter was the G peak, which is attributable to the A1 g vibration mode that is characteristic of sp^3^ defects. The I_D_/I_G_ value of the EG (ca. 0.72) was lower than that of reduced GO, which indicated that the EG had fewer defects than GO, ensuring good electroconductivity [24]. The XRD pattern of the EG is displayed in Figure 3c, which shows a narrow diffraction peak at the 2θ value of 26.42°, corresponding to the (002) plane with a d-spacing of 0.34 nm, indicating that few oxygen groups were introduced between the interlayers of the EG (Figure 3c) [24]. This result suggests that the EG had an oxidized surface but an unoxidized interior, which was beneficial to the self-assembly of the EG and PANI while ensuring good electric conductivity in the final composite. XPS analysis was also used to investigate the chemical components and states of the EG/PANI film (Figure 3e). A significant N peak could be observed in the XPS spectrum of the EG/PANI film, and an additional peak at 285.4 eV (corresponding to C–N species) appeared in the C 1s spectrum of the EG/PANI film, indicating that the PANI had been successfully composited with the EG [25]. As shown in Figure 3e, the high-resolution N 1s spectrum of the EG/PANI film could be fitted with two peaks, corresponding to the pyridinic N (400.2 eV) and pyrrolic N (399.3 eV) species, respectively. The above results were further confirmed with FTIR analysis. As shown in Figure 3f, after introducing PANI, the characteristic peaks of PANI were observed at 1581 and 1494 cm^−1^, corresponding to the vibration peaks of the ring stretching of quinoid and benzenoid forms, respectively. Moreover, the C–N stretching of aromatic amine was located at 1300 cm^−1^, the 1,4-substituted benzene ring was located at 798 cm^−1^, and the asymmetrical stretching vibration of C–H in the pyranoid ring was located at 2924 cm^−1^ [26]. The XPS and FTIR analyses demonstrated the effective compositing of EG and PANI. The conductive EG matrix with PANI active sites would enable the EG/PANI film to show good performance as an electrode for a capacitor [25].

**Figure 4:**
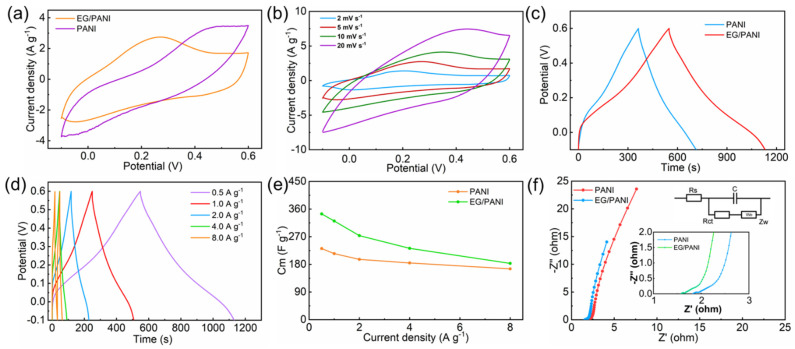
Electrochemical performance in three−electrode configurations: (**a**) CV curves of the PANI and EG/PANI electrodes at a scan rate of 5 mV s^−1^. (**b**) CV curves of the EG/PANI electrode at various scan rates. (**c**) GCD curves of the PANI and EG/PANI electrodes at a current density of 0.5 A g^−1^. (**d**) GCD curves of the EG/PANI electrode at various current densities. (**e**) Gravimetric capacitances of the EG/PANI and PANI electrodes at different current densities. (**f**) EIS measurements with a frequency range of 0.1 Hz to 1 MHz (inset bottom: the EIS curves in the high−frequency range; inset top: equivalent circuit of the Nyquist curve).

The capacitive performance of the EG/PANI electrodes was then studied using three-electrode configurations. The EG/PANI films were directly used as free-standing electrodes without any binders or current collectors. Figure 4a shows the CV curves of the PANI and EG/PANI electrodes at a scan rate of 5 mV s^−1^ and an operating voltage range from −0.1 to 0.6 V (vs. saturated calomel electrode). It was found that the commercial PANI electrode showed significant redox peaks around 0.4 and 0.2 V, exhibiting typical pseudocapacitive behavior [27]. After compositing, the EG/PANI electrode also showed typical pseudocapacitive behavior, indicating that the PANI maintained the activity in the EG/PANI electrode [27]. In addition, the area of the CV curve for the EG/PANI electrode was much larger than that of the PANI electrode, indicating that the EG/PANI electrode showed increased capacitance compared to the PANI electrode. Figure 4b shows the CV curves of the EG/PANI electrode at various scan rates. At a scan rate of 2 mV s^−1^, a high capacitance of 368 F g^−1^ was achieved (equivalent to 268.6 F cm^−3^). This value was higher than that of the PANI electrode (333 F g^−1^ and 100.0 F cm^−3^). Due to the simple casting method without any binders or current collectors, the normalized capacitance of the EG/PANI electrode was much higher than those of previous reported electrodes [28].

GCD measurements at different current densities were further conducted to investigate the capacitance and cycling performance of the electrodes. Figure 4c shows the GCD curves of the PANI and EG/PANI electrodes at a current density of 0.5 A g^−1^. It was found that both the PANI and EG/PANI electrodes showed the feature of pseudocapacitance, demonstrating discharging times of 347.1 s and 582 s, respectively. Figure 4d shows the GCD curves of the EG/PANI electrode at various current densities. The EG/PANI electrode exhibited a higher capacitance (344.3 F g^−1^) than the PANI electrode (231.6 F g^−1^) at the current density of 0.5 A g^−1^, which was consistent with the CV result. Even at 8 A g^−1^, the GCD curve of the EG/PANI electrode still showed pseudocapacitive behavior with a capacitance retention of 53.2% (Figure 4e). The higher capacitance and better rate performance of the EG/PANI composite was due to the higher electric conductivity and the presence of more N/O-containing functional groups. The rich N/O-containing functional groups not only provided numerous active sites for ion storage but also enhanced the wettability of the electrode with regard to the electrolyte.

Since the charge transfer and ion diffusion at the electrolyte/electrode interface are important for capacitive performance [29], electrochemical impedance spectroscopy (EIS) measurements were used to investigate the charge transfer and ion diffusion of the PANI electrode and EG/PANI electrode. As shown in Figure 4f, the EIS curves of the PANI electrode and EG/PANI electrode all showed semicircles in the high- and middle-frequency regions and straight lines in the low-frequency region, reflecting the charge transfer and ion diffusion, respectively. It should be noted that the EG/PANI electrode had a lower equivalent series resistance (ca. 1.6 Ω) than the PANI electrode (ca. 1.8 Ω), confirming that the EG could endow the EG/PANI electrode with good electric conductivity. In the high-frequency region, the EG/PANI electrode also showed a smaller semicircle than the PANI electrode, indicating faster charge transfer for the EG/PANI electrode. Moreover, the nearly vertical lines in the low-frequency region implied that the PANI electrode and EG/PANI electrode had fast ion-diffusion speed and ideal capacitive behavior. The EG/PANI electrode had a straighter line than the PANI electrode in the low-frequency region, indicating faster ion diffusion within the EG/PANI electrode as well [26].

**Figure 5:**
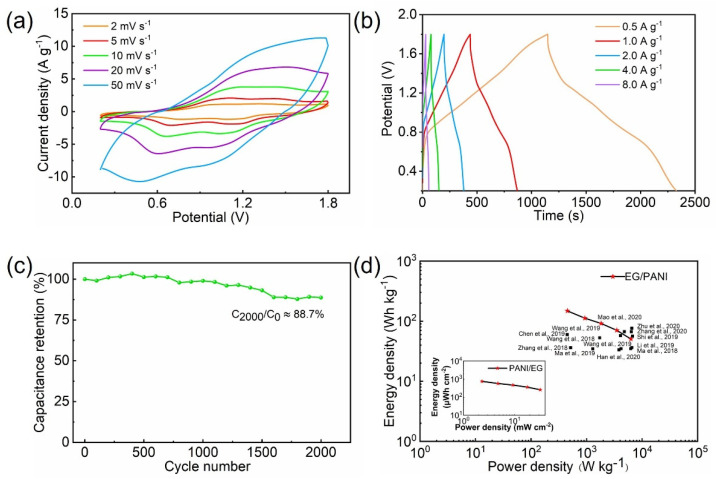
Electrochemical performance of ZMSC using the EG/PANI positive electrode and Zn negative electrode: (**a**) CV curves at different scan rates. (**b**) GCD curves at different current densities. (**c**) Cycling stability at a current density of 5 A g^−1^. (**d**) Ragone plots of the EG/PANI−based ZMSC in comparison to reported results [6,13,33,34,35,36,37,38,39,40,41,42,43].

The EG/PANI film and Zn foil were processed into intergrading electrodes and respectively employed as positive and negative electrodes to assemble the ZMSC. Figure 5a shows the CV curves of the ZMSC at various scan rates from 2 to 50 mV s^−1^ and with an operating voltage range from 0.2 to 1.8 V, exhibiting redox peaks around 1.2 and 0.8 V that indicated typical pseudocapacitive behavior due to the PANI [30]. The capacitances of the ZMSC were calculated to be 324.2, 219.8, 198.0, 164.6, and 104.1 F g^−1^ (corresponding to 1.7, 1.1, 1.0, 0.9, and 0.6 F cm^−2^ and 218.7, 160.4, 144.6, 120.2, and 76.0 F cm^−3^) at 2, 5, 10, 20, and 50 mV s^−1^, respectively. In addition, the capacitive performance of the ZMSC was also measured using GCD curves (Figure 5b) at current densities from 0.5 to 8 A g^−1^ (equivalent to 2.55 to 40.8 mA cm^−2^). According to the GCD curves, the capacitances of the ZMSC were 361.3, 283.8, 244.4, 213.9, and 189.7 F g^−1^ (corresponding to 1.8, 1.4, 1.2, 1.1, and 1.0 F cm^−2^ and 263.7, 207.2, 178.4, 156.1, and 138.5 F cm^−3^) at 0.5, 1, 2, 4, and 8 A g^−1^, respectively. It should be noted that the areal capacitance of the ZMSC based on the EG/PANI electrode (1.8 F cm^−2^) was much higher than those of reported ZMSCs using other positive electrodes (such as MnO_2_, AC, and graphene electrodes, which had low capacitances from 0.001 to 0.5 F cm^−2^) [31]. Moreover, these previous studies usually did not account for the weight of the current collectors, binders, and conductive agents [32]. Commonly, the weight of the binders and conductive agents accounts for ~20% of the total weight of the electrodes. The weight of the current collectors (e.g., copper foams, nickel foams, stainless steel foils) accounts for the majority of the total weight of the electrodes. In addition to high capacitance, the EG/PANI-based ZMSC showed good cycling stability. As shown in Figure 5c, high capacitance retention of 88.7% was obtained at 25.5 mA cm^−2^ (5 A g^−1^) even after 2000 cycles.

We then calculated the energy and power densities based on the areal and volumetric capacitances to evaluate the functional performance of the ZMSC. As shown in the areal Ragone plots (Figure 5d), the ZMSC could reach a high areal energy density of 755.8 μWh cm^−2^ at 2.3 mW cm^−2^ (equivalent to 108.2 Wh L^−1^ at 329.7 W L^−1^ and 148.2 Wh kg^−1^ at 451.7 W kg^−1^). Furthermore, the ZMSC could still deliver an energy density of 257.0 μWh cm^−2^ (36.8 Wh L^−1^ and 50.4 Wh kg^−1^) even at the high power density of 32.0 mW cm^−2^ (5.8 kW L^−1^ and 6.3 kW kg^−1^). The performance of the EG/PANI-based ZMSCs was superior to most of the reported MSCs, such as graphene-based MSCs [33,34], multifaceted microporous carbon MSCs [6,35,36], activated carbon MSCs [37,38,39], MXenes-based MSCs [13,40,41], and metal oxide MSCs [42,43]. It should also be noted that these reported MSCs were usually constructed with current collectors (such as Ni foams, Cu foils, and graphite papers), which commonly have thicknesses of 30~100 μm and weights of 10~40 mg cm^−2^ [44]. If these current collectors were considered, the improvement in the functional performance of our EG/PANI-based ZMSCs would be greater compared to the reported results.

## 3. Materials and Methods

### 3.1. Materials

Preparation of the EG/PANI films: We first prepared EG sheets using a modified electrochemical exfoliation method. Briefly, 1 M (NH_4_)_2_SO_4_ aqueous solution was used as the electrolyte, and 0.1 wt% H_2_O_2_ was added in the electrolyte to adjust the pH value. After that, two graphite flake electrodes of the same shape and area (4 cm in length, 1 cm in width, and about 2 mm in thickness) were placed opposite each other in the electrolytic cell, and the electrodes were immersed in the 1 M (NH_4_)_2_SO_4_ electrolyte. Using a switching power supply (±10 V) and an adjustable electrode distance (3–30 cm), the exfoliated graphene flakes were rapidly stripped, and then the stripped EG flakes were collected and washed via vacuum filtration. The EG precipitate was further dissolved in N-N dimethylformamide solution and ultrasonicated for 0.5 h to uniformly disperse it in the solution. Then, the solution was poured into a centrifuge tube and centrifuged at 3000 r min^−1^ for 30 min. The upper layer of liquid was removed after centrifugation and cleaned with deionized water 5 times to remove the residual electrolyte. Finally, the filter cake obtained by filtration was freeze-dried to obtain EG. Then, the EG sheet and polyaniline (mass ratio 1:1) were dispersed in deionized water and sonicated for 2 h, which was followed by vigorous stirring for 12 h to obtain a uniformly dispersed slurry. The configured EG/PANI slurry was cast on the PET substrate and scraped flat with a 1 mm high squeegee. Then, the squeegee-coated film was put into a drying oven with the temperature set at 35 °C, and the drying time was 2~3 h. Finally, the dried film could be easily peeled off from the substrate by hand to form a self-supporting EG/PANI film.

Process for the EG/PANI-based ZMSCs: ZMSCs were constructed using the interdigital EG/PANI and Zn metal electrodes with the PVA/ZnCl_2_ gel electrolyte. For the preparation of the PVA/ZnCl_2_ gel electrolyte, 2 g of PVA was added into 20 mL de-ionized water, and then 10 mL of 8 M ZnCl_2_ solution was added. The mixture was heated to 90 °C under stirring until it became clear. The designed EG/PANI electrode was simply cut into an interdigitated shape (with a length of 3 cm, width of 1.5 cm, fork tine width of 0.1 cm, fork tine distance of 0.4 cm, thickness of 70 μm, areal density of 5.1 mg cm^−2^, and volume density of 0.73 g cm^−3^) using a designed stamp or laser-engraving machine. The Zn metal electrodes were assembled with elongated Zn foils (with a thickness of 30 μm).

### 3.2. Characterization

The morphologies of the materials were characterized by scanning electron microscopy (SEM) on a ZEISS Sigma 300. The thickness of the EG was determined using an atomic force microscope (AFM) on a Veeco dimension V. X-ray photoelectron spectroscopy (XPS) was conducted on a Thermo Scientific K-Alpha instrument (Waltham, MA, USA) to analyze the nitrogen and carbon atoms within the EG and the EG/PANI composite. The XPS peak-fitting program XPSPEAK 4.1 was used for the spectra processing. Raman spectra were obtained with a Raman system (Renishaw, UK) using a 514.5 nm laser as the light source to characterize the EG. X-ray diffraction (XRD) measurements were examined using a Smart lab 9 kW with Cu-Kα radiation (λ = 0.15406 nm) to analyze the crystal structure of EG. Fourier-transform infrared (FTIR) spectroscopy was performed on a Thermo Scientific Nicolet iS20 instrument to confirm the structure of the EG/PANI composite.

### 3.3. Electrochemical Measurements

Electrochemical measurements were performed using a CHI760E electrochemical workstation. The specific capacitance of the single electrode ($C_{m}$) was measured with three-electrode configurations. In the three-electrode configuration, an EG/PANI film with an area of 1 cm × 1 cm was used as the working electrode. A saturated calomel electrode was used as the reference electrode. A Pt electrode was used as the counter electrode. The electrolyte used in the three-electrode configuration was 1 M H_2_SO_4_ aqueous solution. $C_{m}$ was calculated using the following equations [45]:

(2) $$C_{m}=\frac{\int ΙdV}{2\times s\times \Delta V\times m_{e}}\;(\mathrm{CV}\;\mathrm{method})$$

(3) $$C_{m}=\frac{2I\int Vdt}{m_{e}\times \Delta V^{2}}\;\;\;\;\;(\mathrm{GCD}\;\mathrm{method})$$

where ∫ΙdV is the integrated area under the cyclic voltammetry (CV) curve, s (V s^−1^) is the scan rate, $m_{e}$ (g) is the mass of the single working electrode, ΔV (V) is the scanned potential window, and Ι (A) is the discharge current. GCD is the abbreviation of galvanostatic charge discharge.

The areal capacitance of the MSC device ($C_{M-a}$) were measured with two-electrode configurations. In the two-electrode configuration, the designed EG/PANI electrode was used as the cathode and the elongated Zn foil was used as the anode for the MSC device. $C_{M-a}$ was calculated using the following equations:

(4) $$C_{M-a}=\frac{\int ΙdV}{2\times s\times \Delta V\times a}\;(\mathrm{CV}\;\mathrm{method})$$

(5) $$C_{M-a}=\frac{2I\int Vdt}{a\times \Delta V^{2}}\;\;(\mathrm{GCD}\;\mathrm{method})$$

where *a* is the area of the electrodes.

The areal energy density ($E_{M-a}$) and areal power density ($P_{M-a}$) of the MSC device were calculated based on the GCD results:

(6) $$E_{M-a}=\frac{C_{M-a}\times {\left(V-V_{drop}\right)}^{2}-0.2^{2}}{2\times 3.6}$$

(7) $$P_{M-a}=\frac{E_{M-a}\times 3600}{\Delta t}$$

(8) $$E_{M-m}=\frac{C_{M-m}\times {\left(V-V_{drop}\right)}^{2}-0.2^{2}}{2\times 3.6}$$

(9) $$P_{M-m}=\frac{E_{M-m}\times 3600}{\Delta t}$$

Similar to the calculation of the three-electrode configurations, the specific capacitance of the MSC device ($C_{M-m}$) was obtained based on the mass of the positive electrode of the MSC. The gravimetric energy density ($E_{M-m}$) and gravimetric power density ($P_{M-m}$) of the MSC device were calculated using Equations (8) and (9).

## 4. Conclusions

Due to the unique composition, free-standing ability, and facile electrode patterning of the EG/PANI film, we were able to demonstrate a facile method to prepare high-performance ZMSCs using an EG/PANI positive electrode in a gel electrolyte. The fabricated ZMSCs exhibited ultrahigh areal capacitance of 1.8 mF cm^−2^ at 2.55 mA cm^−2^ (361.3 F g^−1^ at a current density of 0.5 A g^−1^) and a landmark areal energy density of 755.8 μWh cm^−2^ at 2.3 mW cm^−2^ (148.2 Wh kg^−1^ at 451.7 W kg^−1^). We believe this study could provide a facile preparation method for high-performance ZMSC electrodes for the potential integration of miniaturized intelligent electronics.

## Data Availability

Data is contained within the article.

## Sample Availability

Samples available from the authors.
